# Mortality in severely injured children: experiences of a German level 1 trauma center (2002 – 2011)

**DOI:** 10.1186/1471-2431-14-194

**Published:** 2014-07-30

**Authors:** Carsten Schoeneberg, Marc Schilling, Judith Keitel, Manuel Burggraf, Bjoern Hussmann, Sven Lendemans

**Affiliations:** 1 Department of Trauma Surgery, University Hospital Essen, University Duisburg-Essen, Hufelandstraße 55, Essen, Germany

**Keywords:** Trauma, Severely injured, Errors, Pediatric, Mortality

## Abstract

**Background:**

Trauma in pediatric patients is a major cause of death. This study investigated differences between decedents and survivors. Furthermore, an analysis of preventable and potential preventable trauma deaths was conducted and errors in the acute trauma care were investigated.

**Methods:**

All patients aged less than 16 years with an Injury Severity Score (ISS) ≥ 16 upon primary admission to the hospital between July 2002 and December 2011 were included in this study. Decedents were compared with survivors and an analysis of deceased children for preventable and potential preventable deaths was conducted. The acute trauma care was investigated regarding errors in treatment.

**Results:**

Significant differences were found in Glasgow Coma Scale, Injury Severity Score, Revised Trauma Score, New ISS, Revised Injury Severity Classification, and Trauma and Injury Severity Score. Decedents had a worse head trauma with associated coagulopathy. The overall mortality rate was 13.4%. The majority of death occurred soon after arrival. No long term intensive care unit stay was found.

No preventable but one potential preventable death was analyzed. Most errors occurred in fluid volume management and in a delay of starting the therapy for hemorrhage and coagulopathy.

Prolonged preclinical rescue time and surgery time within the first 24 hours was found.

**Conclusions:**

Head trauma is the determinant factor for mortality in severely injured pediatric patients. Death occurred shortly after arrival and long term intensive care stays might be an exception. In treatment of severely injured children volume management, hemorrhage and coagulopathy management, rescue time, and total surgery time should receive more attention.

## Background

Trauma remains a major cause of death for children worldwide [1]. In recently published studies, pediatric patients had a better outcome with a reduced mortality when treated in specialized pediatric trauma centers compared to adult trauma centers [2,3].

The available literature on the treatment of severely injured patients refers mainly to adults. Most of the available algorithms have been transferred from adults to children, but only a few studies focus on severely injured children. Hussmann et al. reported a tendency toward a clinical decline with higher mortality after excessive prehospital fluid replacement [4].

Analyses of errors in the treatment of severely injured patients have been frequently performed in adults [5-8]. The results have led to the development of regionalized trauma care. Furthermore, these studies helped to reduce errors and improve the quality of trauma care.

According to Do et al., children represent only about 10% of all trauma admissions [9]. Together with the unique characteristics of pediatric injuries, treatment of severely injured children can be challenging.

Very few analyses of preventable or potentially preventable deaths in pediatric trauma care are available in the literature. Dykes et al. reported a 13% rate of preventable deaths. They define preventable deaths as all injuries with an Abbreviated Injury Scale (AIS) less than 6, AIS head score of less than 5 and an Injury Severity Score (ISS) of less than 59 [10]. Diamond et al. reported a rate of preventable deaths of 7% for the region of Ontario, Canada [11]. This was a follow-up study of the same region and with the same criteria as the study by Dykes et al.

Esposito et al. reported an overall rate of preventable deaths in children of 9%, their rate for in-hospital deaths was 16% [12].

Only analyses done in North America were available. To the best of our knowledge, this is the first analysis of preventable deaths in Europe.

The aim of our study was to analyze the mortality rates of severely injured children admitted to a level 1 trauma center in Germany between 2002 and 2011. Additionally, all decedents were analyzed regarding errors in their treatment.

## Methods

To examine mortality in severely injured children, data, collected for the Trauma Registry of the German Society for Trauma Surgery (DGU), were analyzed. The data for this registry is collected prospectively.

Additionally, the patients’ hospital records, including the prehospital emergency physician notes, trauma room documentation, operative reports, imaging, electronic labs, and in-patient charts were retrospectively analyzed.

Analysis of the data from the Trauma Registry of the DGU received the full approval of the Ethics Committee of the University of Witten/Herdecke, Cologne, Germany. In addition, full approval of the Ethics Committee of the University of Duisburg-Essen was received.

This analysis was a single-center study of a level 1 trauma center in Germany. Serving the catchment area of the Ruhr district with approximately 5.1 million habitants, it is one of the largest trauma centers in Germany. There are four level 1 trauma centers in the Ruhr district. According to the Whitebook of the DGU, a definition of recommendations on structure, organization, installations, and equipment of trauma centers in Germany, our trauma center fulfills all criteria for a level 1 and pediatric trauma center [13].

Inclusion criteria were primary admission to the hospital, an Injury Severity Score (ISS) ≥ 16, admission between 1st July 2002 and 31st December 2011, and age of up to and including 15 years. No exclusion was made for deaths on arrival or in the trauma room.

The types of information collected of the included patients are presented in Table 1.

**Table 1:** Collected information of included patients

**Scales**	**General information**	**Laboratory test**	**Time periods**	**Interventions**
Injury Severity Score (ISS)	Age	First hemoglobin value	Time from admission to cranial computed tomography (CCT) and to whole-body CT	Intubation at AS and in TR
Abbreviated Injury Scale (AIS)	Gender	Initial number of platelets	Time in trauma room	Resuscitation at AS and in TR
New ISS	Systolic blood pressure at AS and in TR	Partial Thromboplastin time (PTT)	Preclinical rescue time	Thoracic drainage at AS and in TR
Glasgow Coma Scale (GCS)	Heart rate at AS and in TR	Prothrombin time	Length of intensive care unit (ICU) stay	
Revised Trauma Score (RTS)	Oxygen saturation at AS and in TR	Base excess		
Revised Injury Severity Classification (RISC)	Count of performed surgery	Lactate		
Trauma and Injury Severity Score (TRISS)	Administered fluid volume			
	Multiple organ failure (MOF)			
	Sepsis			
	Type of injury (penetrating vs. blunt)			

AS, Accident scene; TR, Trauma room.

For the analysis of errors occurring in treatment, James Reason’ the definition of an error - “the failure of a planned action to achieve its desired goal” - formed the basis of the identification of errors [14]. Furthermore, the recommendations of the DGU guideline “Treatment of Patients with Severe and Multiple Injuries” and the European guideline “Management of bleeding and coagulopathy following major trauma” were used for error analysis [15,16]. For judging errors in time periods we used the average time of all treated patients in our hospital. The average time for preclinical rescue was 45 minutes, for trauma room treatment 30 minutes, and for the first emergency surgery 120 minutes [17].

All deaths were classified into one of three categories: non-preventable, potentially preventable, and preventable. MacKenzie published three criteria for a potentially preventable death: the injury must be survivable, the delivery of care is suboptimal, and the error in care must be directly or indirectly implicated in the death of the patient [18]. Shackford et al. defined preventability as followed: “(1) Anatomic injury or combination of injury considered survivable; (2) Physiologic state at time of arrival of first responder critical to judgment of preventability; patient generally stable; if unstable patient becomes stable with treatment; (3) Evaluation and management suspect in any way” [19]. Additionally, the definition of the WHO described in the “Guidelines for trauma quality improvements programs” was applied [20].

Every death was judged individually by every author as non-preventable, potentially preventable, or preventable. When opinions differed, the case was discussed until consensus was reached.

Data were analyzed with the Statistical Package for the Social Sciences (version 21; SPSS: An IBM Company; Chicago, IL, USA). Incidences were presented as percentages, measured values as means and standard deviation (SD) in cases of continuous variables and for categorical variables the median and the interquartile range (IQR) were included. Differences were evaluated using the Chi-squared test in cases of categorical variables; and the t-test in cases of continuous variables. When performing the t-test, a Levene-test was also performed. In cases of variance heterogeneity, the Welch-test was used instead of the t-test. When an obvious deviation from normality was found, continuous variables were tested with a non-parametric rank test (Mann–Whitney). Normal distribution was tested using the Kolmogorow-Smirnow-test. Data was considered significant at p < 0.05.

## Results

Between 1st July 2002 and 31st December 2011, a total of 2304 patients were admitted to the hospital. Of those, 256 (11.1%) were children 15 years old and younger. A total of 82 patients had an ISS ≥ 16 and were primarily admitted to hospital.

The mortality rate was 13.4%. The median ISS was 25, the median GCS was 9, the mean age was 7.4 years, and the expected death rate, represented by the RISC score, was 20.3%. The characteristics of the study population are reported in Table 2. The cause of accident is presented in Table 3. The most common cause of injury was traffic accident as a pedestrian, followed by falls from a height of above 3 meters.

**Table 2:** Characteristics of the study population

	**Median**	**Interquartile range**	**Mean value**	**Standard deviation**
**ISS**	25	11		
**AIS Head**	4	2		
**AIS Thorax**	1	3		
**AIS Extremities**	0	3		
**AIS Skin**	0	1		
**GCS**	9	11		
**RISC**			20.3	30.5
**TRISS**			0.8	0.3
**Age**			7.4	5.0

GCS, Glasgow Coma Scale; ISS, Injury Severity Score; RISC, Revised Injury Severity Classification; TRISS, Trauma and Injury Severity Score; AIS, Abbreviated Injury Scale.

**Table 3:** Cause of injury in the study population

**Cause of injury**	**Percentage**
Traffic accident, car	12.2%
Traffic accident, motorcycle	2.4%
Traffic accident, bicycle	4.9%
Traffic accident, pedestrian	26.8%
Fall > 3 m	19.5%
Fall < 3 m	14.6%
Suicide	2.4%
Others	17.2%

Table 4 shows the differences between decedents and survivors. Significant differences are indicated with an asterisk.

**Table 4:** Differences between non-survivors and survivors

	**Non-Survivors**	**Survivors**	**p value**
**Patients (n)**	11	71	
**ISS**	38 (41)	22 (12)	< 0.001*
**AIS Head**	5 (0)	4 (2)	< 0.001*
**AIS Face**	0 (2)	0 (0)	= 0.181
**AIS Thorax**	2 (4)	0 (3)	= 0.487
**AIS Abdomen**	0 (3)	0 (0)	= 0.337
**AIS Extremities**	0 (3)	0 (3)	= 0.911
**AIS Skin**	0 (0)	0 (1)	= 0.013*
**New ISS**	57 (28)	26 (12)	< 0.001*
**GCS**	3 (0)	10 (10)	< 0.001*
**RTS**	2.0 ± 2.7	6.5 ± 1.5	< 0.001*
**RISC**	74.7 ± 26.2	12.1 ± 21.4	< 0.001*
**TRISS**	0.2 ± 0.4	0.9 ± 0.2	< 0.001*
**Age**	7.3 ± 4.4	7.4 ± 5.1	= 0.951
**SBP AS**	70 (115)	110 (33)	= 0.012*
**Heart rate AS**	55 (106)	105 (30)	= 0.010*
**Oxygen saturation (%) AS**	75 (196)	95 (18)	= 0.086
**SBP TR**	80 (119)	108 (32)	= 0.048*
**Heart rate TR**	84 (137)	106 (33)	= 0.332
**Oxygen saturation (%) TR**	98 (24)	99 (2)	= 0.216
**Number of surgery**	2 (3)	1 (2)	= 0.352
**Pre-hospital volume (ml)**	695.5 ± 735.0	794.3 ± 852.3	= 0.564
**TR volume (ml)**	2073.9 ± 2289.6	739.9 ± 584.9	= 0.094
**Total volume (ml)**	2923.9 ± 2963.1	1460.1 ± 1011.2	= 0.210
**Hb (g/dl)**	8.6 ± 2.8	10.6 ± 1.8	= 0.014*
**Platelets (gpt/l)**	139.6 ± 72.1	257.9 ± 91.6	< 0.001*
**PTT (sec.)**	108.3 ± 48.9	27.8 ± 8.5	< 0.001*
**Prothrombin time (%)**	45.8 ± 23.3	82.4 ± 19.8	< 0.001*
**Base excess**	-4.9 ± 10.9	-4.9 ± 5.2	= 0.741
**Lactate (mmol/l)**	13.1 ± 16.4	2.3 ± 3.8	= 0.021*
**Time from admission to CCT (min)**	33.2 ± 11.8	31.6 ± 11.4	= 0.722
**Time from admission to whole-body CT (min)**	35.0 ± 12.0	29.4 ± 10.6	= 0.467
**Time in TR (min)**	39.4 ± 23.1	51.7 ± 19.2	= 0.114
**Preclinical rescue time (min)**	44.1 ± 16.5	42.9 ± 19.9	= 0.976
**ICU stay (days)**	2 (3)	5 (12)	= 0.001*
**Hospital stay (days)**	2 (2)	13 (16)	< 0.001*
**Gender Male**	72.7%	70.4%	= 0.876
**Rate of whole-body CT**	57.1%	57.4%	= 0.989
**MOF**	85.7%	23.2%	= 0.001*
**Sepsis**	33.3%	14.7%	= 0.235
**Intubation at AS**	100%	61.4%	= 0.012*
**Resuscitation at AS**	45.5%	7.4%	< 0.001*
**Thoracic drainage at AS**	0%	5.9%	= 0.455
**Intubation/Re-intubation/Change to tracheal tube in TR**	55.6%	43.9%	= 0.511
**Resuscitation in TR**	37.5%	4.6%	= 0.001*
**Thoracic drainage in TR**	25.0%	4.6%	= 0.029*
**Emergency Surgery**	75.0%	30.9%	= 0.014*
**Penetrating trauma**	0%	0%	
**Suicide**	0%	2.8%	= 0.573

Categorical variables are presented as median with the interquartile range in parentheses, continuous variables as means with ± standard deviation, and incidences as percentages; *significant differences; GCS, Glasgow Coma Scale; RTS, Revised Trauma Score; ISS, Injury Severity Score; RISC, Revised Injury Severity Classification; TRISS, Trauma and Injury Severity Score; AIS, Abbreviated Injury Scale; SBP, Systolic blood pressure; AS, Accident scene; TR, Trauma room; Hb, Hemoglobin; PTT, Partial Thromboplastin time; ICU, Intensive care unit; CCT, Cranial computed tomography; MOF, Multi-organ failure.

Deceased children were significantly more seriously injured (ISS 38 vs. 22) than the survivors. Also, the expected mortality, represented by the TRISS and the RISC, was statistically different. The non-survivors suffered from more serious head injuries with an obviously increased AIS head score (5 vs. 4) and decreased GCS (3 vs. 10).

In the group of the non-survivors, the systolic blood pressure and the heart rate at the accident scene were significantly lower (70 vs. 110 mmHg; 55 vs. 105 bpm). At arrival in the trauma room, the systolic blood pressure remained decreased in the non-survivors (80 vs. 108 mmHg), whereas the heart rate no longer showed significant differences.

All coagulation parameters (platelets, PTT, prothrombin time) were worse in deceased children. Additionally, these children had signs of bleeding with reduced hemoglobin of 8.6 g/dl compared with 10.6 g/dl in survivors.

Lactate was significantly lower in survivors (2.3 vs. 13.1 mmol/l), while the base excess showed no difference.

No statistical differences were found for the administered fluid volume, but a trend was verified for higher fluid volume in the non-survivors.

In the group of non-survivors, the rate of multi organ failure was higher (85.7% vs. 23.2%) the rate of sepsis was not statistically higher, but the trend was towards higher rates in deceased children (33.3 vs. 14.7%).

All non-survivors were intubated at the accident scene (survivors 61.4%) and five of eleven patients received cardio-pulmonary resuscitation (survivors 7.4%) No deceased child received thoracic drainage at the accident scene (survivors 5.9%), but 25% of this group needed thoracic drainage in the trauma room (survivors 4.6%). 75% of the non-survivors underwent an emergency surgery, as did 30.9% of the survivors.

All of the children who died suffered from severe head injuries with an AIS head score 5 or 6. No preventable deaths occurred. One death was designated as potentially preventable (Patient 11), although the AIS head was 5, because of an accumulation of errors. The initial intubation was not possible and it lasted 20 minutes to establish a secured airway. The child suffered from hemorrhage, but the initial recognized coagulopathy was not treated for 10 hours. Additionally, a time delay of 106 minutes to surgical treatment occurred and the first surgical treatment lasted 175 minutes. Therefore, this patient was declared as potentially preventable. The patients’ characteristics are presented in Table 5.

**Table 5:** Characteristics of the non-survivors

**Patient**	**GCS**	**ISS**	**RISC**	**AIS Head**	**AIS Neck**	**AIS Thorax**	**AIS Abdomen**	**AIS Extremities**	**AIS Skin**	**Age**
**1**	3	66	78.2	5	1	5	4	0	0	5
**2**	3	38	56.7	5	0	2	0	3	1	0
**3**	3	38	69.8	5	2	2	3	0	0	7
**4**	3	59	94.1	5	3	5	0	2	0	15
**5**	3	75	99.9	6	0	0	0	0	0	7
**6**	3	25	%	5	0	0	0	0	0	7
**7**	3	25	97.7	5	0	0	0	0	0	7
**8**	9	25	15.4	5	0	0	0	0	0	15
**9**	3	29	65.7	5	2	0	0	0	0	4
**10**	3	75	100.0	5	0	4	5	5	0	5
**11**	3	50	69.2	5	3	4	0	3	0	8

% not available; GCS, Glasgow Coma Scale; ISS, Injury Severity Score; RISC, Revised Injury Severity Classification; AIS, Abbreviated Injury Scale.

The RISC Score of Patient 8 demonstrated a very high probability of survival. But the patient suffered a severe subdural hematoma and a subarachnoid hemorrhage. A short period after surgical treatment the patient died.

**Figure 1:**
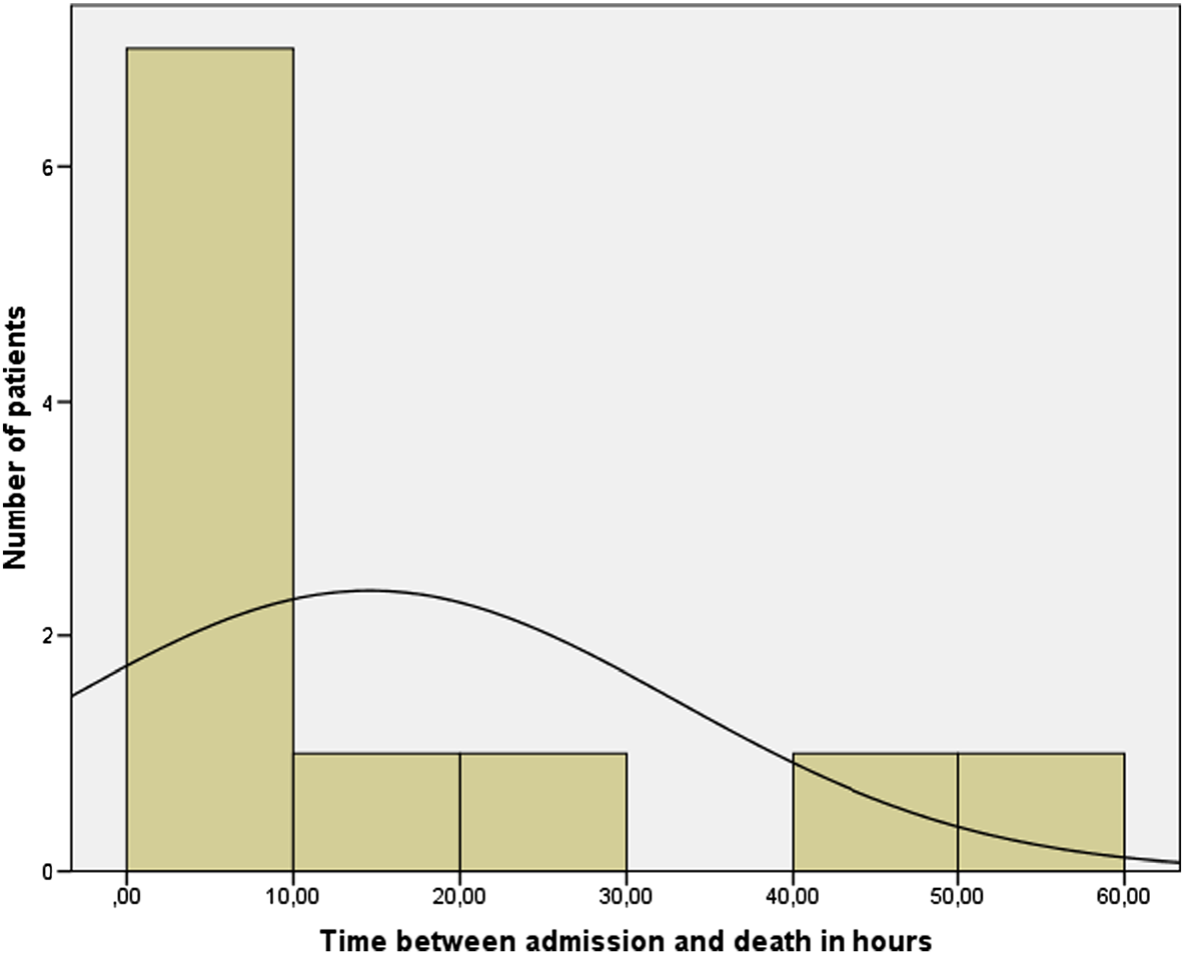
Time between admission and death; Mean value 14.58 h; standard deviation: 18.4; N = 11.

Although no death was classified as preventable, an error analysis was performed. Five children received a fluid volume of more than 1500 ml. In one child, intubation was not possible at the scene until 20 minutes after responders arrived. In three children, no coagulation medication was substituted, so one child had insufficient coagulation for 10 hours. In one patient, the first surgical treatment lasted 175 minutes and in another a second surgical intervention was necessary in the first 24 hours, because of increased cerebral swelling after initial decompressive craniotomy. In one child, no concentrated red cells were available in the trauma room, although signs for bleeding existed and so the first transfusion was performed two-and-a-half hours after arrival. In one patient, the intubation had to be redone in the trauma room because of bleeding. In three children, the time from accident to hospital was longer than 60 minutes.Only three of the patients who died survived the first day after admission. Figure 1 show the time of death and the average time of survival after admission.

## Discussion

Analyzing trauma deaths according to preventable and the analysis of errors occurring in the treatment of trauma patients are common. These studies are helpful to improve trauma care. A lot of studies analyzing preventable deaths and errors were performed in adults [5-8], but only a few focused on pediatric patients. Our study, in contrast to others, included only severely injured children. Moreover, to avoid the influence of inter-hospital transfer, we only included patients, who were primary admitted to our hospital.

The Trauma Registry of the DGU, one of the largest trauma registries worldwide, does not include the data, which is necessary to perform analyses of preventable deaths and occurred errors in the treatment of severely injured patients. Only differences between non-survivors and survivors of documented data in the Trauma Register could be analyzed, for example the administered fluid volume. Therefore, a single-center study is the only way to perform a preventable death and error analyses in Germany.

Because of these system related limitations, our including criteria, and the small number of severely injured pediatric patients, we were only able to include 82 patients. However, our results supported, that errors occur in the treatment of pediatric trauma patients, and therefore, analyzing preventable deaths and errors in children might be as important as in adults.

Similar to the findings of Do et al. [9] the rate of pediatric trauma admissions in our hospital was 11%. Of these admissions, 32% were severely injured with an ISS ≥ 16. The mortality rate was lower than suspected, represented by the RISC score of 20.3% and the mortality rate was lower than in adults during the same period (13.4% vs. 28.7%) [17].

Analyzing the differences between non-survivors and survivors revealed that non-survivors were more severely injured with a lower GCS and higher AIS head score. The AIS head score was the only AIS score that differed between the groups. So it seems that more serious head trauma is the determining injury for mortality in children. This is analog to the results of other studies [21-24].

Non-survivors presented a coagulopathy. One possible explanation is that acute trauma-associated coagulopathy is triggered by brain injury [25,26]. This is explained by the expression of tissue thromboplastin and tissue factor from the injured brain [27-29].

In the literature, a more severe base excess is associated with higher mortality [30]. This finding could not be validated in this analysis, because there was no significant difference between the two groups.

The lactate was significantly higher in the group of non-survivors. According to Hindy-Francois et al., this is associated with higher mortality [31].

In our study, the infused total fluid volume was not significantly higher in non-survivors than in survivors, but there was a trend toward higher volume in deceased children. This is according to Hussmann et al. [4], who reported worse outcomes for children receiving excessive fluid volume. One possible explanation for administration of higher fluid volume is that non-survivors had a lower systolic blood pressure and a reduced heart rate at arrival. This might be caused by hemorrhage but another explanation might be that these findings were mis-interpreted as hemorrhage, because it is known that severe head trauma can be associated with a lower systolic blood pressure and reduced heart rate because of auto regulation dysfunctions. This dysfunction is associated with a higher mortality rate [32,33].

In our study, children died very soon after trauma. Only three patients survived the first day. There was no hospital stay greater than 52 hours. In adults, a trimodal mortality model was described [34]. This model could not be validated, although, because of only 11 non-survivors, a definitive conclusion is not possible.

According to the definitions used by Dykes [10] and Diamond [11], no preventable death occurred in the study population because all non-survivors suffered serious head trauma with an AIS head score of 5 or 6. However, errors in treatment of severely injured children occurred.

As mentioned above, massive fluid volume infusion might be associated with a higher mortality rate. In five of eleven deaths in this study, the infused volume was greater than 1500 ml, and even the only zero years old child received a total of 8500 ml. However, because of a lack of guidelines according the volume therapy in pediatric trauma patients a cut-off, for when the infused volume is excessive, is impossible to define.

Damage control surgery is a principle in the treatment of adult patients [35]. In one child, the emergency surgery lasted 175 minutes, because of a craniofacial reconstruction. This is against the principle of damage control surgery, and so it was defined as an error. Another child needed a second surgery within the first 24 hours. In an analysis of adults who died after trauma, a longer surgery time within the first 24 hours was associated with a worse outcome [36].

To the best of our knowledge, this is the first study in Germany investigating preventable deaths in pediatric trauma patients.

### Limitations

There are some limitations of this study. First of all, it is a retrospective analysis, although the data from the Trauma Registry of the DGU are collected prospectively. Second, this is a single-center study. Third, only a small number of deaths occurred in children during the time period of the study. Therefore, our analyses are limited because of the small number of patients. However, the hospital is one of the largest in Germany, so only an analysis of the entire Trauma Registry of the DGU would provide a greater number of patients. But the data of the Trauma Registry support no analysis of preventable deaths and only a limited error analysis. No information on pre-hospital mortality of children was obtained, since no data were available.

## Conclusion

Brain trauma is the determinant factor in mortality in severely injured pediatric patients. This injury is associated with acute trauma-associated coagulopathy. The overall trauma mortality in children is lower than that of adults. If children die after a severe injury, it happens soon after the trauma. In the study population, long-term intensive care unit stays were not found. Therefore, the known trimodal mortality model of adults might not be suitable in pediatric trauma care.

No preventable death was found in this analysis. However, errors in the acute care of injured children were evident. A great number of the children received an excessive infusion of fluid volume, which might be associated with higher mortality. Also, errors in control of hemorrhage were found. Additionally, extended preclinical rescue time and surgery time within the first 24 were found. Optimizations in these fields might further reduce mortality.

## Abbreviations

AIS: Abbreviated injury scale; CCT: Cranial computed tomography; DGU: German society for trauma surgery; GCS: Glasgow coma scale; Hb: Hemoglobin; ICU: Intensive care unit; ISS: Injury severity score; MOF: Multi-organ failure; PTT: Partial thromboplastin time; RISC: Revised injury severity classification; RTS: Revised trauma score; SD: Standard deviation; SPSS: Statistical package for the social sciences; TRISS: Trauma and injury severity score; vs: Versus.

## Competing interests

The authors declare that they have no competing interests.

## Authors’ contributions

CS and SL designed this study. CS, MS, JK, MB and BH collected and analyzed the data. CS drafted the manuscript, and all authors contributed substantially to its revision. CS takes responsibility for the paper as a whole. All authors read and approved the final manuscript for publication.

## Pre-publication history

The pre-publication history for this paper can be accessed here:

http://www.biomedcentral.com/1471-2431/14/194/prepub
